# Troponin for surveillance of immune checkpoint inhibitor–associated myocarditis and post-therapy cardiovascular risk stratification: a systematic review and meta-analysis

**DOI:** 10.3389/fcvm.2026.1800066

**Published:** 2026-06-26

**Authors:** Ahmed J. Alaraibi, Mustafa Alani, Layla Al-Nooh, Nairouz Quateen, Lulwa Al-Qallaf, Retaj Al-Azemi, Mohammed Taqi, Abdullatif Alfehaid, Paul Murray

**Affiliations:** 1 School of Medicine, Royal College of Surgeons in Ireland-Medical University of Bahrain, (RCSI-MUB), Busaiteen 228, Bahrain; 2 Department of Pathology, Royal College of Surgeons in Ireland-Medical University of Bahrain (RCSI-MUB), Busaiteen 228, Bahrain

**Keywords:** cardiotoxicity, immune-checkpoint inhibitors, immune-related adverse events, MACE, myocarditis, troponin, meta-analysis

## Abstract

**Background:**

Immune checkpoint inhibitors (ICIs) have transformed cancer therapy but are associated with immune-related adverse events, including myocarditis, a rare complication with high morbidity and mortality. ICI-associated myocarditis often presents with nonspecific clinical features, making early recognition challenging. Cardiac troponin is a widely available biomarker of myocardial injury and is increasingly used in cardio-oncology surveillance pathways; however, its performance for identifying ICI-associated myocarditis and its prognostic value for subsequent cardiovascular outcomes remain incompletely defined.

**Objectives:**

To evaluate the performance of cardiac troponin in the surveillance and identification of ICI-associated myocarditis and to assess its prognostic value for major adverse cardiovascular events (MACE) and mortality.

**Methods:**

We conducted a systematic review and meta-analysis in accordance with PRISMA 2020 guidelines. Medline (PubMed), Embase, Cochrane CENTRAL, and Scopus were searched from inception to October 2025 for diagnostic and prognostic studies evaluating troponin (I or T; conventional or high-sensitivity) in adults receiving ICIs. Random-effects models were used to pool effect estimates. Performance for identifying ICI-associated myocarditis was assessed using odds ratios (ORs), and prognostic associations were summarized using hazard ratios (HRs).

**Results:**

Across diagnostic studies, troponin elevation was associated with confirmed ICI-associated myocarditis (pooled log OR 4.14, 95% CI 2.80–5.48), with no significant between study heterogeneity. However, false-positive troponin elevations were common, resulting in modest positive predictive value despite high sensitivity. In prognostic analyses, elevated troponin levels were associated with an increased risk of MACE (pooled HR 6.14, 95% CI 3.61–10.45) and all-cause mortality (pooled HR 1.95, 95% CI 1.36–2.81). These associations were consistent across studies with varying designs, troponin assays, and outcome definitions.

**Conclusions:**

Troponin elevation in patients receiving ICIs is associated with ICI-related myocarditis and with increased risk of adverse cardiovascular outcomes and mortality, supporting its role as an accessible biomarker for early clinical evaluation, surveillance, and risk stratification. However, because false-positive elevations are common and diagnostic performance varies by assay type, threshold, and timing, troponin should not be interpreted as a standalone diagnostic test. Prospective studies are needed to define optimal troponin-guided surveillance strategies.

**Systematic Review Registration:**

https://www.crd.york.ac.uk/PROSPERO/view/CRD420251176007 identifier CRD420251176007.

## Introduction

The discovery of immune checkpoint inhibitors (ICIs), including programmed cell death protein-1 (PD-1), programmed death-ligand 1 (PD-L1), and cytotoxic T-lymphocyte–associated antigen4 (CTLA-4) inhibitors, has marked a major advance in cancer immunotherapy (1). These agents enhance anti-tumor immune responses by targeting inhibitory immunologic receptors on T lymphocytes, thereby reinvigorating host immune surveillance against malignancy (1). An expanding number of indications now support the use of ICIs across a broad range of cancers in both curative and palliative settings (2). Despite their clinical success, ICIs are associated with immune-related adverse events (irAEs), among which myocarditis represents one of the most severe and potentially fatal complications (3). Although the reported incidence of ICI-associated myocarditis is relatively low, the condition carries substantial morbidity and mortality, often presents with nonspecific symptoms, and poses significant diagnostic challenges (4). Cardiac troponins (I and T), including high-sensitivity assays, are established biomarkers of myocardial injury and are widely used for diagnosis and risk stratification in acute coronary syndromes. Their role in the detection of ICI-associated myocarditis and in predicting cardiovascular outcomes has received increasing attention. Elevated troponin concentrations may reflect subclinical myocardial inflammation, and serial measurements have been proposed as a surveillance strategy during ICI therapy (5). Contemporary cardio-oncology guidelines recommend baseline cardiovascular assessment before initiation of ICI therapy, including clinical evaluation, electrocardiography, and cardiac biomarkers such as troponin. In patients with suspected ICI-associated myocarditis, current guidance supports prompt multimodality evaluation incorporating serial biomarkers, electrocardiography, echocardiography, cardiac magnetic resonance imaging (CMR), and selected endomyocardial biopsy (EMB) (38). However, recommendations regarding routine serial troponin surveillance remain variable because of limited prospective evidence and uncertainty regarding optimal thresholds, timing, and interpretation. However, despite its incorporation into current diagnostic frameworks, the extent to which troponin elevation aligns with established diagnostic reference standards, such as cardiac magnetic resonance imaging (CMR), endomyocardial biopsy (EMB), or consensus clinical definitions (e.g., Bonaca or IC-OS), remains incompletely characterized. Similarly, the prognostic value of troponin elevation for predicting adverse cardiovascular outcomes including major adverse cardiovascular events (MACE), cardiac death, heart failure, and all-cause mortality has not been systematically established. Prior studies have demonstrated associations between troponin elevation and ICI-related cardiotoxicity, but findings remain heterogeneous owing to variability in study design, patient populations, assay platforms, and outcome definitions.

Accordingly, this systematic review and meta-analysis was undertaken to address two key objectives: first, to evaluate the diagnostic performance of cardiac troponin for detecting ICI-associated myocarditis in adult patients with cancer, using established reference standards such as CMR, EMB, or predefined consensus clinical criteria; and second, to assess the prognostic value of troponin elevation after ICI initiation for predicting adverse cardiovascular outcomes, including MACE, cardiac death, heart failure, and all-cause mortality, in adults receiving ICI therapy.

## Methods

This systematic review and meta-analysis was conducted in accordance with the Preferred Reporting Items for Systematic Reviews and Meta-Analyses (PRISMA) 2020 guidelines. The protocol was registered with the International Prospective Register of Systematic Reviews (PROSPERO), under the registration ID: CRD420251176007.

### Eligibility criteria

Studies were selected according to predefined double PICO criteria:

Population:

Adults (≥18 years) with any cancer type receiving ICI therapy (anti– PD-1, anti–PD-L1, or anti–CTLA-4 agents), evaluated either for suspected ICI-associated myocarditis or for cardiovascular risk stratification following ICI initiation.

Index Test/Prognostic Factor:

Cardiac troponin I or T (conventional or high-sensitivity assays) measured during or after ICI therapy, with clearly defined or derivable thresholds.

Comparator:

For diagnostic analyses: Independent reference standards for myocarditis, including cardiac magnetic resonance imaging (Lake Louise criteria), endomyocardial biopsy, or prespecified clinical consensus definitions.

For prognostic analyses: Normal troponin levels as defined by assay-specific thresholds.

Outcomes:

Diagnostic outcomes included confirmed ICI-associated myocarditis and diagnostic accuracy metrics (sensitivity, specificity, predictive values, likelihood ratios, diagnostic odds ratio, and AUC).

Prognostic outcomes included major adverse cardiovascular events (MACE), cardiac death, new or worsening heart failure, overall cardiovascular events, and all-cause mortality. Eligible study designs included prospective or retrospective cohort studies, cross-sectional diagnostic accuracy studies, and nested case–control studies. Case reports, small case series (<10 patients), pediatric or animal studies and non–ICI-related myocarditis, were excluded.

### Information sources and data collection process

Medline (PubMed), Embase, Cochrane Central Register of Controlled Trials (CENTRAL), and Scopus were systematically searched from database inception to October 2025 to identify eligible studies evaluating the diagnostic and prognostic performance of cardiac troponin in adults receiving ICIs. Only English-language studies were included. The search was restricted to English-language publications because translation resources were not available, and accurate extraction of diagnostic thresholds, myocarditis adjudication criteria, and time-to-event estimates required detailed full-text interpretation. Detailed search strategies are provided in Appendix 1.

Additional studies were identified through manual screening of reference lists from relevant reviews and included articles. All citations were imported into Covidence, and duplicates were removed electronically and manually prior to screening.

Two investigators, Layla Al-Nooh (LM) and Nairouz Quateen (NQ), independently screened titles and abstracts. Full-text articles were reviewed by Layla Al-Nooh (LM), Nairouz Quateen (NQ), Ahmed Alarabi (AA), and Lulwa Al-Qallaf (LA). Disagreements were resolved through discussion or consultation with a Mohammed Taqi (MT). Data were extracted using a standardized electronic form. For diagnostic studies, extracted variables included troponin assay type and thresholds, numbers of troponin-positive cases, confirmed myocarditis cases, and 2 × 2 diagnostic table components. For prognostic studies, extracted data included troponin values, timing of measurement, clinical presentation, incidence of MACE or mortality, and reported effect estimates.

Risk of bias was assessed using QUADAS-2 for diagnostic studies and QUIPS for prognostic studies. Certainty of evidence was evaluated using the GRADE framework.

### Outcomes

The primary diagnostic outcome was confirmed ICI-associated myocarditis, defined using cardiac magnetic resonance imaging, endomyocardial biopsy, or prespecified clinical consensus criteria. Diagnostic performance was assessed using sensitivity, specificity, and odds ratios derived from troponin positivity at defined thresholds.

The primary prognostic outcome was major adverse cardiovascular events (MACE), defined as a composite of cardiovascular death, myocardial infarction, myocarditis-related complications, malignant arrhythmias, cardiogenic shock, heart failure hospitalization, or need for mechanical circulatory support, according to study-specific definitions.

Secondary prognostic outcomes included all-cause mortality, cardiac death, overall cardiovascular events, and new or worsening heart failure. When multiple outcome definitions were available, the most comprehensive composite endpoint was selected.

Effect estimates were extracted as hazard ratios, risk ratios, or odds ratios. When not directly reported, estimates were derived from available event counts and follow-up data. When multiple troponin assays or thresholds were reported, the assay and threshold most aligned with contemporary clinical practice were selected for primary analysis.

### Data synthesis

Meta-analyses were performed using the *meta* and *metafor* packages on R. Between-study heterogeneity was assessed using the I^2^ statistic with 95% confidence intervals. For diagnostic studies, effect sizes were pooled as log odds ratios. For prognostic analyses, hazard ratios were extracted or derived, log-transformed, and synthesized using random-effects inverse-variance methods. Three dichotomous prognostic outcomes were evaluated: major adverse cardiovascular events (MACE), cardiovascular death, and all-cause mortality. A random-effects model using inverse-variance weighting was applied to all meta-analyses, as between-study heterogeneity was anticipated given differences cancer type, ICI regimen, troponin assay platform, positivity threshold, timing of measurement, reference standard, and outcome definitions.

## Results

### Study selection

A total of 24 studies comprising 7,258 participants met the inclusion criteria and were included in the systematic review. Of these, four studies were eligible for the diagnostic performance meta-analysis. Eleven studies contributed quantitative data to the prognostic analyses and were synthesized in two separate meta-analyses evaluating the association between troponin elevation and major adverse cardiovascular events (MACE) and mortality, respectively.

**Figure 1:**
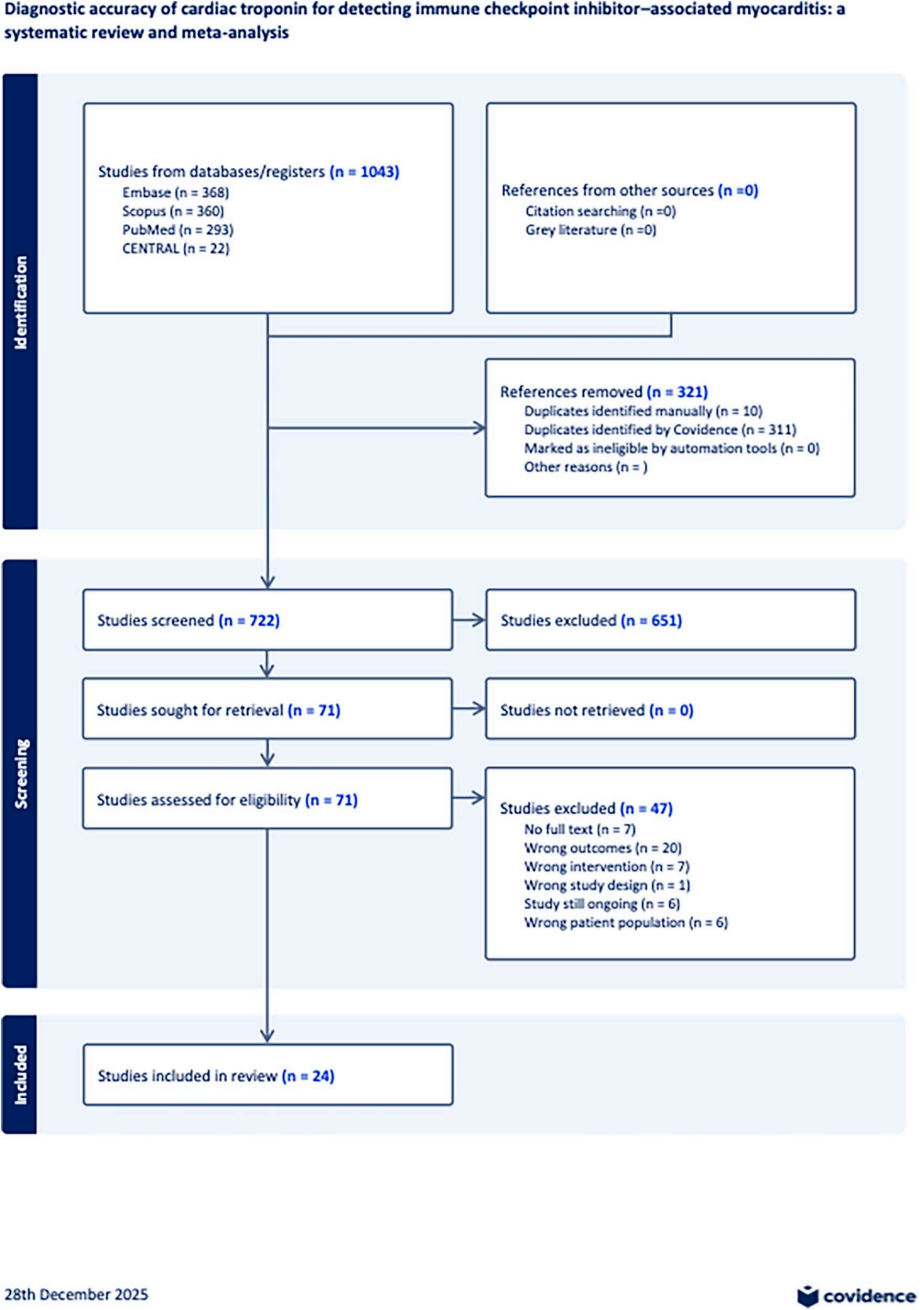
PRISMA flow Diagram of Study Selection This figure summarizes the identification, screening, eligibility assessment, and final inclusion of studies according to PRISMA 2020. Records were retrieved from Medline (PubMed), Embase, Cochrane CENTRAL, and Scopus, with additional studies identified through reference-list screening. After duplicate removal, titles/abstracts and full texts were screened against prespecified criteria. The figure reports reasons for exclusion at fulltext review and the final number of studies included in quantitative meta-analyses and narrative synthesis. PRISMA, Preferred Reporting Items for Systematic Reviews and MetaAnalyses; CENTRAL, Cochrane Central Register of Controlled Trials.

The remaining studies were included in qualitative synthesis only because of insufficient reporting of extractable diagnostic measures or effect estimates, or heterogeneity in outcome definitions. Non-eligible cohorts within included studies were excluded from quantitative analyses. For example, prognostic estimates from the ICI-myocarditis subgroup defined by Bonaca criteria in Pereyra Pietri et al. were not pooled because troponin elevation was not required for diagnosis. The complete study selection process is illustrated in the PRISMA flow diagram (Figure 1).

### Characteristics of included studies

Study characteristics are summarized in Table 1. Publications spanned 2018–2025 and originated from North America, Europe, and Asia. Designs included prospective and retrospective cohorts, observational registries, and case–control analyses. Clinical settings varied and included systematic screening of ICI-treated patients, diagnostic evaluation of symptomatic individuals, and prognostic follow-up after suspected or confirmed ICI-associated myocarditis.

**Table 1:** Characteristics of included studies.

**Study ID**	**Publication _Year_**	**Country**	**Study Design**	**Study Context**	**Cancer Type/s (Most Common)**	**Sample Size (Total)**	**Troponin Type**	**Threshold**	**Reference Standard Used**
Cheng et al. (6)	2025	USA	RCS	Screening	N/A	428	cTnI	≥99th percentile ULN	ESC- ICOS, Bonaca
Furukawa et al. (14)	2023	Japan	PCS	Screening	Mixed (Head/Neck)	126	cTnI	≥99th percentile ULN+≥2× baseline rise	ESC-ICOS
Lehmann et al. (20)	2023	France/Germany	PCS	Diagnostic/Prognostic	Mixed (NSCLC)	60	cTnI/cTnT	≥99th percentile	EMB, Cardiac MRI
Oikawa et al. (11)	2025	Japan	RCS	Screening	Mixed (Gastric)	468	cTnI	ULN; ROC-derived ≥99th percentile ULN	JCS 2023 Guideline
Perelman et al. (23)	2025	Israel	RCS	Prognostic	Mixed (NSCLC)	455	cTnI	ROC-derived threshold	Clinical Diagnosis
Pereyra Pietri et al. (24) (ESC-ICOS Cohort)	2025	USA	RCS	Screening/Prognostic	Mixed (Lung cancer)	47	cTnT	≥99th percentile ULN; ROC-derived	ESC-ICOS, Bonaca
Sarocchi et al. (16)	2018	Italy	PCS	Screening/Prognostic	(NSCLC)	59	cTnI/cTnT	≥99th percentile ULN	Clinical Diagnosis
Shibutani et al. (27)	2025	Japan	RCS	Prognostic	Mixed (Head and Neck)	108	cTnI	≥99th percentile ULN or relative rise	Clinical Diagnosis
Tamura et al. (7)	2022	Japan	RCS	Diagnostic	Mixed (Head and Neck)	129	cTnI	≥99th percentile ULN	ESC-ICOS
Todo et al. (15)	2025	Japan	RCS	Screening	(RCC)	86	cTnI/cTnT	≥99th percentile ULN	ESC-ICOS
Van den Berg et al. (10)	2024	Netherlands	PCS	Screening/Prognostic	Mixed (Melanoma)	164	cTnT	≥99th percentile ULN with significant rise	ESC-ICOS
Vasbinder et al. (13)	2022	USA	OCS	Screening	Mixed (Melanoma)	2606	cTnT	≥99th percentile ULN	Clinical Diagnosis
Zornitzki et al. (12)	2025	Israel	RCS	Screening	Mixed (Lung cancer)	455	cTnI	>50 ng/L	Bonaca
Tomsitz et al. (8)	2025	Germany	PCS	Screening/Diagnostic	Skin Cancers	280	cTnT	≥99th percentile ULN; ROC-derived	ESC-ICOS
Waliany et al. (9)	2021	USA	POS	Screening	Mixed (NSCLC)	214	cTnI	≥99th percentile ULN	Clinical Diagnosis
Zhuang et al. (26)	2024	China	RCS	Prognostic	Mixed (Lung cancer)	45	cTnI	ROC-derived threshold	Bonaca
Guan et al. (25)	2025	China	RCS	Prognostic	Mixed(Esophageal Carcinoma)	90	cTnI	99th Percentile	ESC-ICOS
Power et al. (28)	2024	Multinational	RCS	Prognostic	Mixed (Skin cancers)	748	cTnI/cTnT	Not Specified	Bonaca
Qin et al. (21)	2024	China	ROS	Prognostic	Mixed (Lung cancer)	31	Unspecified	≥ 4× ULN	Clinical Diagnosis
Mahmood et al. (17)	2018	USA	CCS	Prognostic	Mixed (Melanoma)	140	cTnT	Rocderived;study specifc	ESC-ICOS
Chitturi et al. (18)	2019	USA	RCS	Prognostic	Lung cancer	252	cTnI	study-specific	Clinical Diagnosis
Barliz Waissengein et al. (19)	2023	Israel	ROS	Prognostic	Mixed (NSCLC)	71	cTnI	>50 ng/L	ESC-ICOS
Fan et al. (22)	2025	China	RCS	Prognostic	Mixed (Lung cancer)	161	cTnI	≥50× ULN	ESC- ICOS,Bonaca
Dubey et al. (29)	2025	USA	CCS	Prognostic	Mixed (NSCLC)	35	cTnT	99th percentile ULN	ESC-ICOS

USA, United States of America; RCS, retrospective cohort study; PCS, prospective cohort study; OCS, observational cohort study; CCS, case-control study; NSCLC, non-small cell lung cancer; RCC, renal cell carcinoma; cTnI, cardiac troponin i; cTnT, cardiac troponin T; ULN, upper limit of normal; ROC, receiver operating characteristic; ESC-ICOS, european society of cardiology-international cardio-oncology society; JCS, Japanese circulation society; N/A, not applicable/not available.

Patient populations were heterogeneous with respect to cancer type, and sample sizes ranged from small single-center cohorts (<50 participants) to large observational studies (>2,000 participants). Both cardiac troponin I and T assays were used, with increasing adoption of highsensitivity platforms in recent studies. Definitions of troponin positivity and testing protocols varied widely, as did reference standards for myocarditis, which included consensus clinical criteria, guideline-based scores, cardiac magnetic resonance imaging, endomyocardial biopsy, or multimodal clinical assessment. In several studies, troponin elevation formed part of the diagnostic criteria, limiting suitability for inclusion in diagnostic accuracy pooling.

### Diagnostic meta-analysis: diagnostic performance of troponin for ICI-related myocarditis

**Figure 2:**
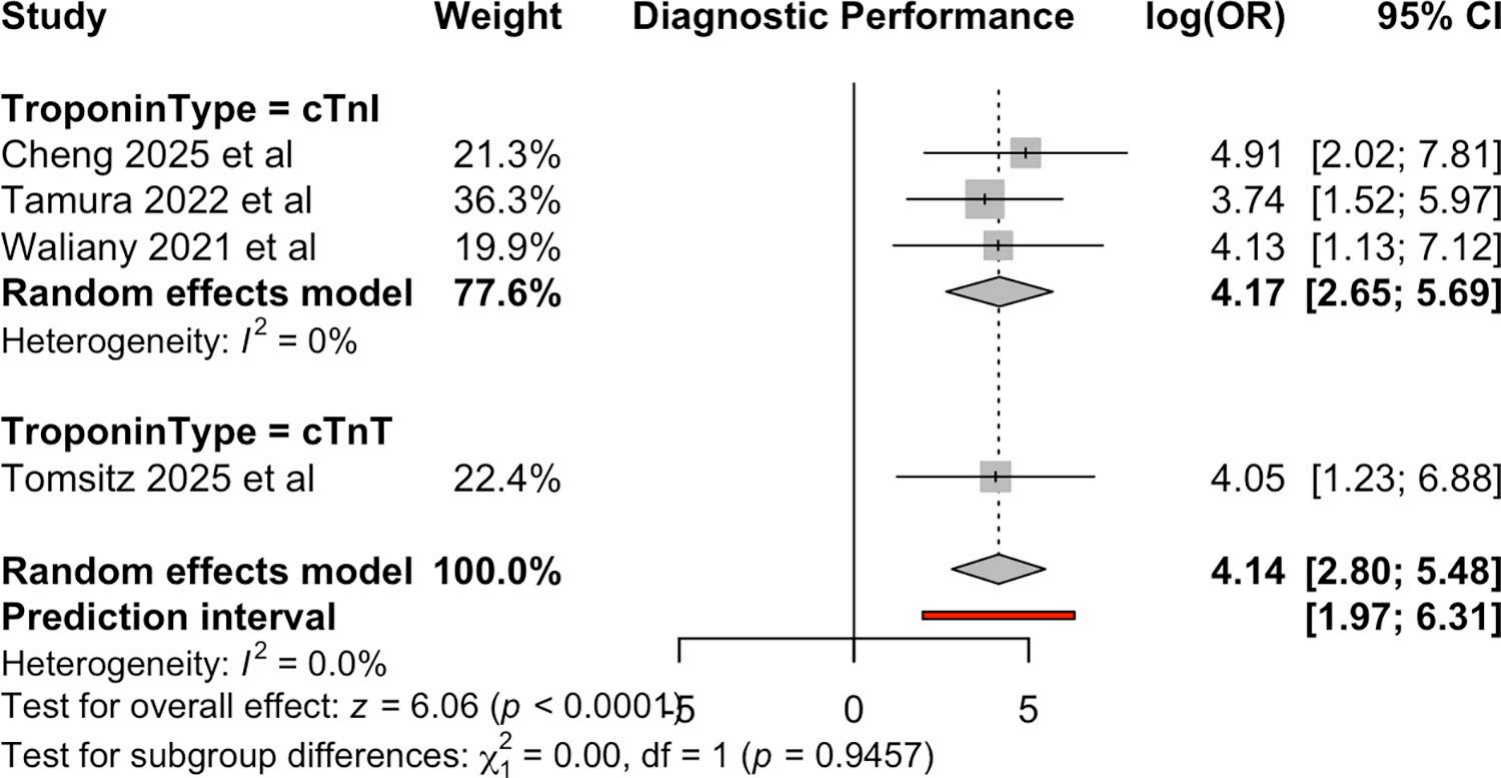
diagnostic meta-analysis forest plot. Forest plot showing study-level and pooled associations between troponin elevation and confirmed immune checkpoint inhibitor (ICI)-associated myocarditis. Effect estimates are presented as odds ratios with 95% confidence intervals, and the pooled estimate was calculated using a random-effects model. Between-study heterogeneity is reported using *I*^2^. ICI, immune checkpoint inhibitor; OR, odds ratio; CI, confidence interval.

Across four diagnostic studies (Cheng et al. (6), Tamura et al. (7), Tomsitz et al. (8), and Waliany et al. (9)), encompassing a total of 1,051 participants, elevated troponin levels were associated with confirmed ICI-associated myocarditis, with a pooled log odds ratio of 4.14 (95% CI 2.80–5.48; *p* < 0.0001). Between-study heterogeneity was negligible (*I*^2^ = 0%). Restriction of the analysis to the three cTnI studies still yielded a strong and consistent association between troponin elevation and confirmed ICI-associated myocarditis, with a log odds ratio of 4.17 (95% CI 2.65–5.69) and no evidence of between-study heterogeneity (*I*^2^ = 0%) (Figure 2).

Across the four diagnostic studies, troponin elevation identified 30 true-positive, 166 false-positive, 1 false-negative, and 850 true-negative cases. Reported sensitivity ranged from 83.3% to 100%, specificity from 74.8% to 91.5%, positive predictive value from 12.5% to 22.7%, and negative predictive value from 92.7% to 100%. These findings suggest high sensitivity and negative predictive value for excluding clinically overt ICI-associated myocarditis, but modest positive predictive value due to frequent false-positive troponin elevations among ICI-treated patients without confirmed myocarditis. A comprehensive description of the diagnostic studies is shown in Table 2.

**Table 2:** Outcomes table for diagnostic/screening studies.

**Study ID**	**Troponin-Positive Cases**	**Myocarditis Cases (Confirmed)**	**Sensitivity (%)**	**Specificity (%)**	**PPV (%)**	**NPV (%)**	**AUC Myocarditis (if available)**	**Time to Onset Myocarditis Median (Days)**
Cheng et al. (6)	42	6	100%	91.5%	14.3%	100%	N/A	56 days
Lehmann et al. (20)	cTnT: 56	60	cTnT: 98.2%	N/A	N/A	N/A	N/A	N/A
	cTnI: 37		cTnI: 88.1%					
Perelman et al. (23)	225	6	56.90%	59.90%	64.%	52.6%	N/A	N/A
Pereyra Pietri et al. (24) ESC-ICOS Cohort	47	47	93.6%	85.4%	83.9%	80.6%	0.93	44 days
Zhuang et al. (26)	48	48	100%	90.30%	12.5%	100%	N/A	42 days
Guan et al. (25)	90	90	N/A	N/A	N/A	N/A	N/A	42 days
Power et al. (28)	711	748	N/A	N/A	N/A	N/A	0.7	40 days
Qin et al. (21)	15	31	69.3%	94.40%	N/A	N/A	0.82	44 days
Mahmood et al. (17)	33	35	N/A	N/A	N/A	N/A	0.81	34 days
Chitturi et al. (18)	22	1	N/A	N/A	N/A	N/A	N/A	N/A
Barliz Waissengein et al. (19)	8	2	N/A	N/A	N/A	N/A	N/A	N/A
Fan et al. (22)	107	161	N/A	N/A	N/A	N/A	0.8	32 days
Dubey et al. (29)	35	35	N/A	N/A	N/A	N/A	N/A	42 days
Tamura et al. (7)	18	6	83.33%	74.8%	22.7%	92.7%	N/A	
Tomsitz et al. (8)	112	16	100%	63.64%	14.3%	100%	N/A	
Waliany et al. (9)	214	3	100%	90.3%	12.5%	100%	N/A	

PPV, positive predictive value; NPV, negative predictive value; AUC, area under the curve; cTnI, cardiac troponin I; cTnT, cardiac troponin T; N/A, not applicable/not available; ESC-ICOS, european society of cardiology-international cardio-oncology society.

### Narrative synthesis of screening and diagnostic studies

Across screening and diagnostic studies, serial cardiac troponin testing during ICI therapy frequently identified troponin elevations, whereas confirmed ICI-associated myocarditis remained uncommon.

In the prospective surveillance study by Waliany et al., routine hs-troponin I testing detected elevations ≥55 ng/L in 11.2% of patients, yet only 1.4% were ultimately diagnosed with myocarditis after multidisciplinary evaluation, indicating that fewer than one in eight troponinpositive patients had myocarditis (9). Similarly, van den Berg et al. reported hs-troponin T elevation above the upper reference limit in 58% of monitored patients; however, only 4.9% met hierarchical diagnostic criteria for definite myocarditis, with most cases classified as hypertroponinemia without myocarditis (10).

In a retrospective cohort with scheduled monitoring, Oikawa et al. observed cTnI elevations in 5.6% of patients, of whom only 15.4% were diagnosed with myocarditis using guideline-based criteria (11). Zornitzki et al. reported elevated hs-troponin I in 11.0% of patients, but only 26% were classified as having probable or possible myocarditis using consensus definitions (12). Large observational data similarly demonstrated that although troponin was universally elevated in confirmed myocarditis cases, the majority of troponin elevations during ICI therapy were attributable to alternative clinical causes (13).

Across studies, patients with confirmed myocarditis consistently exhibited higher absolute troponin concentrations and more pronounced or sustained rises. In van den Berg et al., all definite myocarditis cases had hs-troponin T levels exceeding 160 ng/L, whereas lower-risk groups remained below this range (10). In Waliany et al., the positive predictive value of troponin increased markedly at higher concentrations (≥1,000–2,000 ng/L), while the negative predictive value remained high (9).

### Meta-analysis: troponin and Major adverse cardiovascular events (MACE)

**Figure 3:**
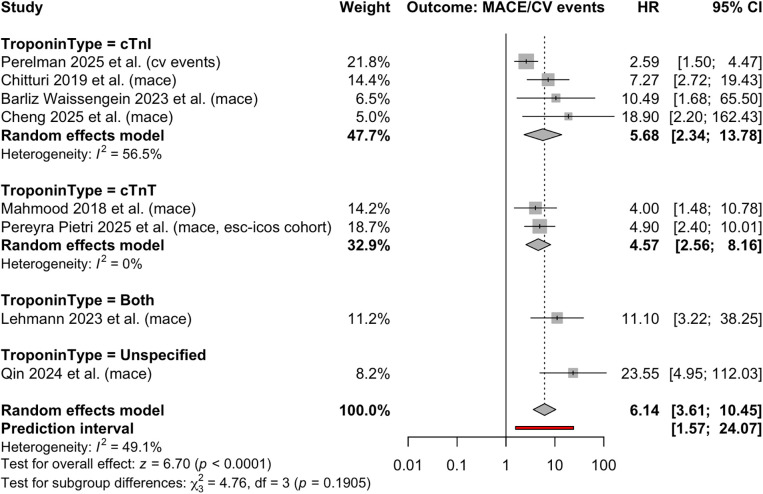
Troponin and MACE forest plot. Forest plot of the prognostic meta-analysis evaluating the association between troponin elevation and major adverse cardiovascular events (MACE) or cardiovascular events in patients receiving immune checkpoint inhibitors. Study-level hazard ratios with 95% confidence intervals are shown, with a pooled random-effects estimate. Statistical heterogeneity is summarized using *I*^2^. MACE, major adverse cardiovascular events; ICI, immune checkpoint inhibitor; HR, hazard ratio; CI, confidence interval.

**Figure 4:**
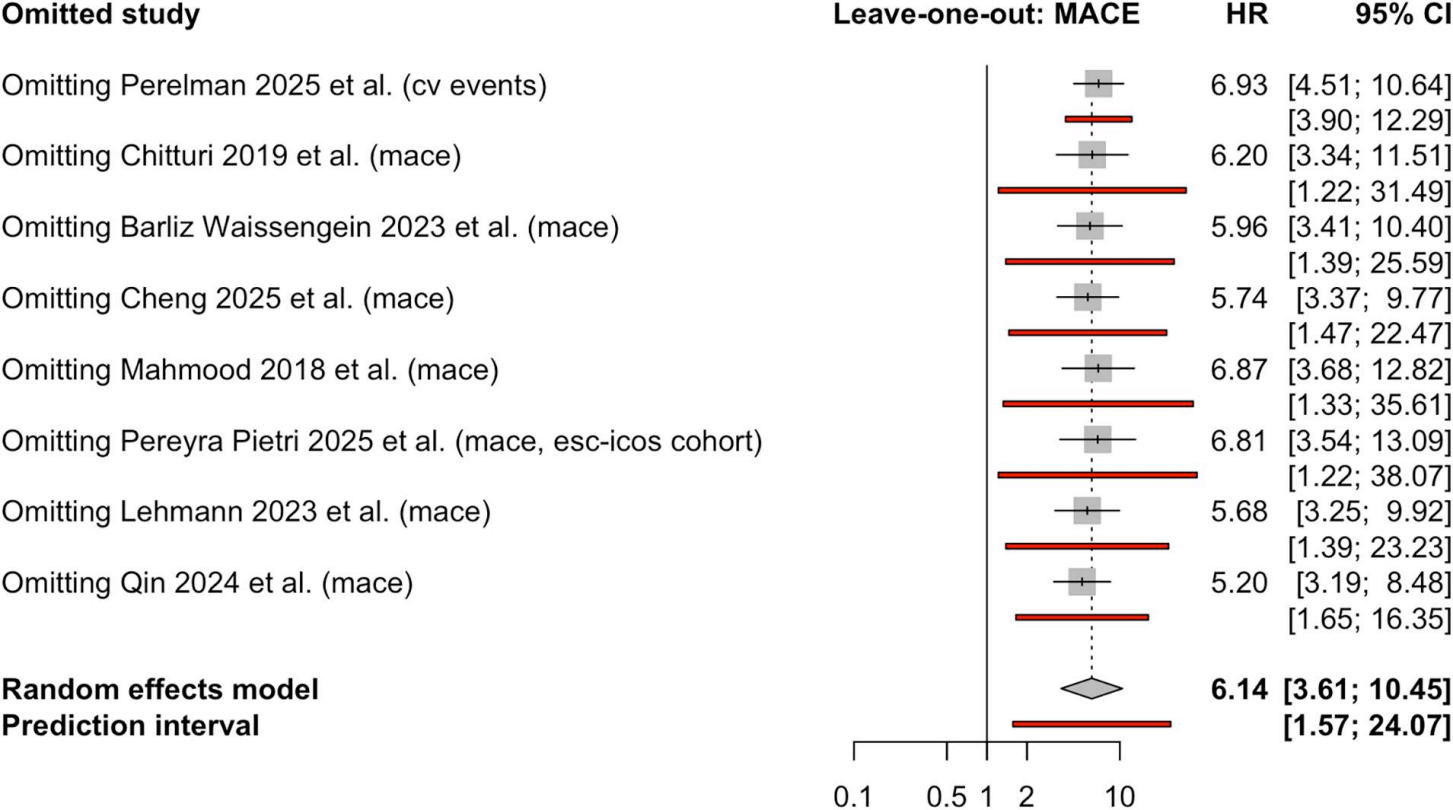
Leave-One-Out sensitivity analysis for troponin and MACE. Leave-one-out sensitivity analysis for the meta-analysis of troponin elevation and MACE/cardiovascular events. Each row shows the pooled hazard ratio recalculated after omitting one study at a time. Consistency of the pooled estimates across iterations indicates that the overall association is not driven by a single study. MACE, major adverse cardiovascular events; HR, hazard ratio; CI, confidence interval.

Eight studies, comprising a total of 1,484 participants, contributed to the quantitative synthesis evaluating the association between troponin elevation and major adverse cardiovascular events (MACE) or cardiovascular events after the initiation of ICI therapy. These included studies by Mahmood et al. (17), Chitturi et al. (18), Waissengrin et al. (19), Lehmann et al. (20), Qin et al. (21), Cheng et al. (6), Gvili-Perelman et al. (23), and Pereyra Pietri et al. (24). In the random-effects meta-analysis, troponin elevation was associated with a markedly increased risk of MACE, with a pooled hazard ratio of 6.14, (95% CI 3.61–10.45). Moderate between-study heterogeneity was observed (*I* = 49.1%, *p* = 0.055), while the overall effect was statistically significant (*z* = 6.7, *p* < 0.0001). Subgroup analyses according to troponin assay type (cTnI, cTnT, both, or unspecified) did not demonstrate statistically significant differences between groups (*χ*^2^ = 4.76, df = 3, *p* = 0.1905). Prediction intervals remained above unity, indicating consistency of the association across study settings (Figure 3). Leave-one-out sensitivity analyses, summarized in Figure 4, showed stable pooled estimates, with hazard ratios ranging from 5.20 to 6.93 following sequential exclusion of individual studies, confirming robustness of the findings. 

### Meta-analysis: troponin and mortality

Eight studies, comprising a total of 1,317 participants, reported mortality outcomes and were included in the quantitative synthesis. These comprised cohorts reported by Guan et al. (25), Zhuang et al. (26), Fan et al. (22), Cheng et al. (6), Lehmann et al. (20), Qin et al. (21), Gvili-Perelman et al. (23), and Pereyra Pietri et al. (24), with outcomes including short-term mortality, all-cause mortality, and cardiac-specific death. In the randomeffects meta-analysis, troponin elevation in cancer patients after receiving ICI treatment was associated with a significantly increased risk of mortality, with a pooled hazard ratio HR 1.95, (95% CI 1.36–2.81). Substantial heterogeneity was observed across studies (*I*^2^ = 80.9%, *p* < 0.0001); however, the overall association remained statistically significant (*z* = 3.59, *p* = 0.0003).

**Figure 5:**
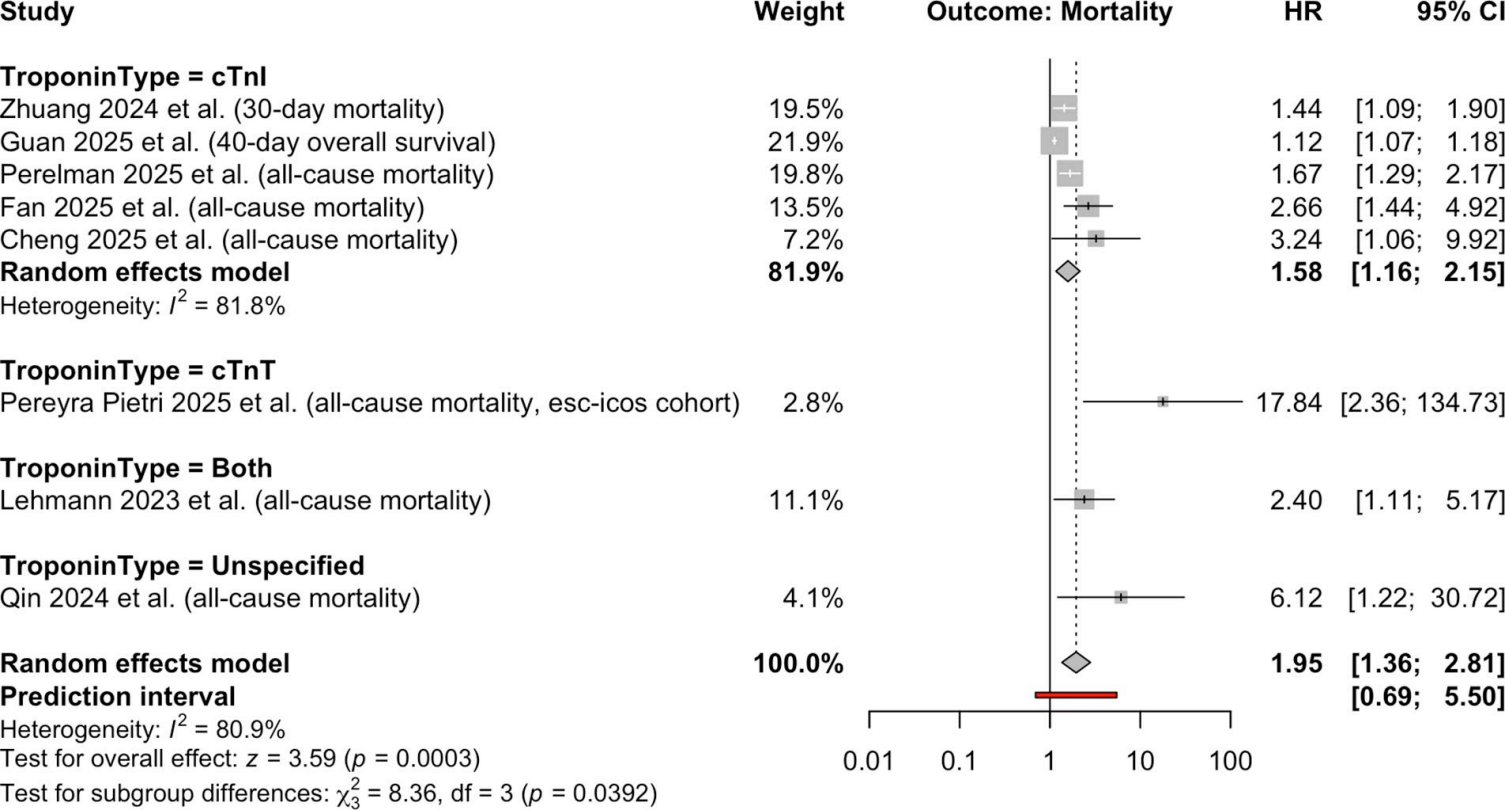
Troponin and mortality forest plot. Forest plot of the prognostic meta-analysis evaluating the association between troponin elevation and mortality in immune checkpoint inhibitor–treated patients. Studylevel hazard ratios with 95% confidence intervals are displayed, and the pooled estimate was generated using a random-effects model. Between-study heterogeneity is reported using *I*^2^. ICI, immune checkpoint inhibitor; HR, hazard ratio; CI, confidence interval.

Subgroup analyses by troponin assay type did demonstrate statistically significant differences (*χ*^2^ = 8.36, df = 3, *p* = 0.0392) (Figure 5).

**Figure 6:**
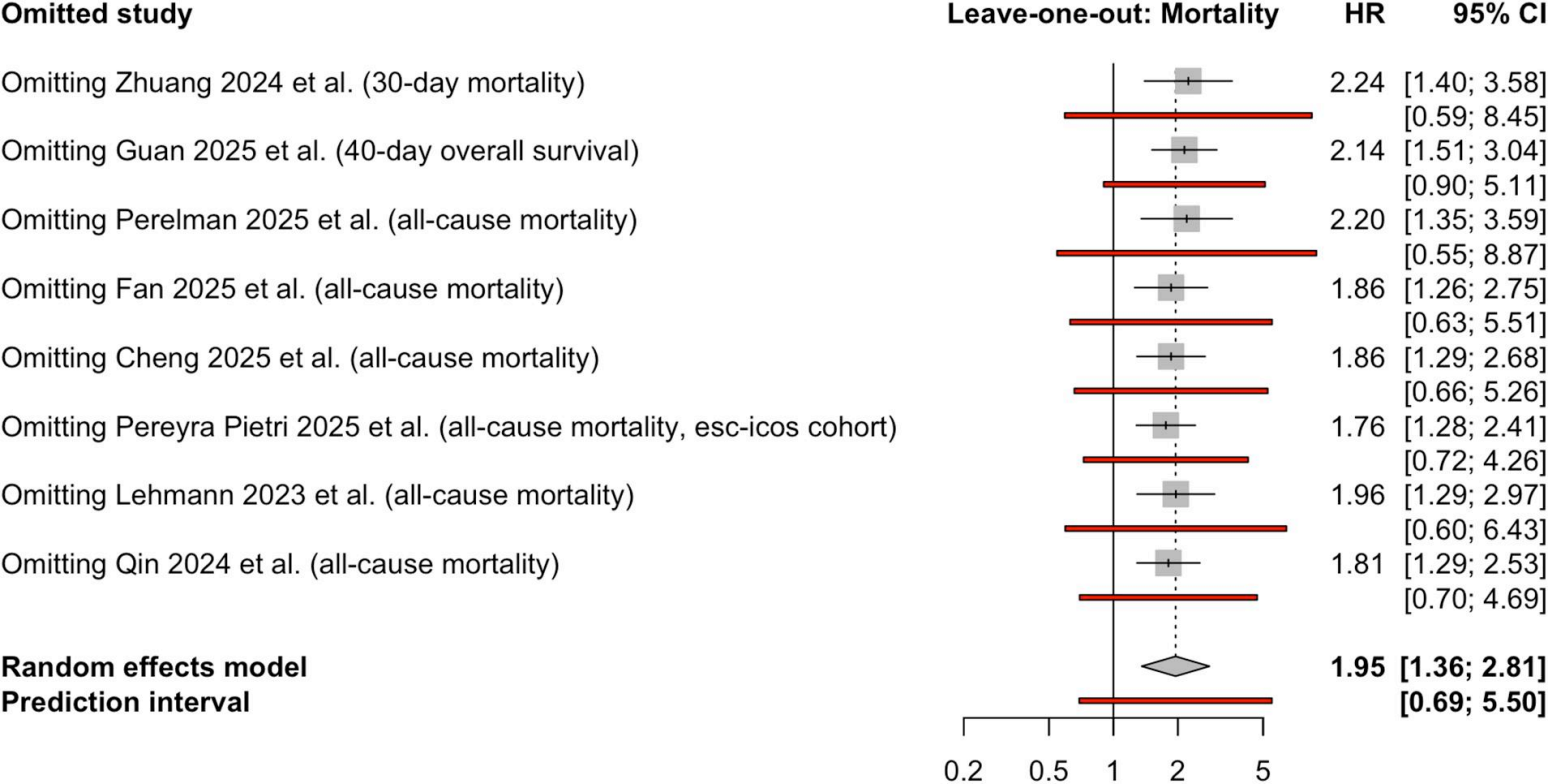
Leave-One-Out sensitivity analysis for troponin and mortality. Leave-one-out sensitivity analysis for the meta-analysis of troponin elevation and mortality. Each row presents the pooled hazard ratio after sequential exclusion of one study. The stability of pooled estimates across exclusions supports robustness of the overall association despite between-study heterogeneity. ICI, immune checkpoint inhibitor; HR, hazard ratio; CI, confidence interval.

Leave-one-out sensitivity analyses, summarized in Figure 6, confirmed the stability of the pooled estimate, with hazard ratios ranging from 1.76 to 2.24 following exclusion of individual studies. Prediction intervals crossed unity, reflecting heterogeneity in effect magnitude across populations and outcome definitions.

### Narrative synthesis of prognostic studies

Across prognostic studies, the magnitude and trajectory of troponin elevation were consistently associated with short-term mortality and adverse cardiovascular outcomes in patients receiving ICIs, particularly among those with ICI-associated myocarditis (17, 26–29).

In a cohort of 45 patients with confirmed myocarditis, Zhuang et al. reported a 30-day mortality rate of 24.4%, with troponin I independently predicting death (HR 1.44, 95% CI 1.09–1.89). A troponin I threshold of 0.87 ng/mL demonstrated good discriminative performance (AUC 0.80) (26). Mahmood et al. similarly observed that higher peak troponin T levels were associated with increased mortality and severe cardiac complications, with fulminant cases exhibiting markedly higher values (17).

Registry data from the International ICI-Myocarditis Registry (*n* = 748) further confirmed a strong dose-esponse relationship between troponin magnitude and adverse outcomes. Patients with troponin levels ≥2,000× the upper limit of normal had a nearly five-fold increased risk of major cardiotoxic events (HR 4.80, 95% CI 2.54–9.08), with 30-day cardiotoxic mortality of 13% (28).

Dynamic changes in troponin also carried prognostic significance. In Dubey et al., a troponin T decline ≥42% within 8 days of corticosteroid initiation was independently associated with improved survival, whereas persistent or rising levels predicted poor outcomes (29).

Several studies demonstrated that troponin elevation predicted major adverse cardiovascular events (MACE) even in the absence of overt myocarditis (18, 19, 28). Elevated troponin I was independently associated with cardiovascular events in lung cancer patients treated with ICIs (18), and hs-troponin I elevation after early treatment cycles predicted subsequent MACE and mortality (19).

Finally, studies in patients without diagnosed myocarditis showed that subclinical troponin elevation was common and prognostically heterogeneous, with higher or persistent elevations associated with worse survival (7, 10, 27). A clearer description of prognostic studies is illustrated in Table 3.

**Table 3:** Outcomes table for prognostic studies.

Study ID	Troponin-Positive Cases	Peak Troponin Median (Range)	Proportion Asymptomatic Elevations (%)	Time to Onset for MACE Median (Days)	Hazard Ratio (HR) MACE	Confidence Interval [95% Ratio CI] Mortality	Hazard (HR)	Confidence Interval (CI)	HR/s Association
Cheng et al. (6)	42	136 ng/L (80–464)	59.5%	56 days	18.9	[2.2–162.5; 95% CI]	3.24	[1.06–9.94; 95% CI]	1. MACE
									2. All-cause
									Mortality
Lehmann et al. (20)	cTnT: 56	cTnT: 40 ng/L (10–70)	27%	5 days	11.1	[3.2–38.0; 95% CI]	2.4	[ 1.1–5.1; 95% CI]	1. All-cause Mortality
	cTnI: 37	cTnI: 12 ng/L (6–64)							
Perelman et al. (23)	225	8 ng/L (5.0, 22.5)	N/A	41 days	2.59	[1.50–4.46; 95% CI]	1.67	[1.29–2.17; 95% CI]	1. MACE
									2. All-cause
									Mortality
Pereyra Pietri et al. (24) ESC-ICOS Cohort	47	670 ng/L (N/A)	N/A	44 days	4.90	[2.40–10.02; 95% CI]	17.84	[2.36–134.62; 95% CI]	1. MACE
									2. All-cause Mortality
Zhuang et al. (26)	48	N/A	33%	N/A	N/A	N/A	1.44	[1.09–1.89; 95% CI]	1. 30 Day Mortality
Guan et al. (25)	90	N/A	N/A	42 days	N/A	N/A	1.122	[1.071–1.176; 95% CI]	1. 40-day
									Overall
									Survival
Qin et al. (21)	15	495 ng/L (N/A)	13%	44 days	23.55	[1.69–38.25; 95% CI]	6.12	[1.22–30.75; 95% CI]	1. MACE
									2. All-cause Mortality
									Mortality
Mahmood et al. (17)	33	2680 ng/L (0.24–7.63)	N/A	34 days	4.0	[1.5–10.9; 95% CI]	N/A	N/A	1. MACE
									2. All-cause Mortality
Chitturi et al. (18)	22	N/A	N/A	36 days	7.27	[2.72–19.43; 95% CI]	N/A	N/A	1. MACE
									2. All-cause Mortality
Barliz Waissengein et al. (19)	8	120 ng/L (72–355)	N/A	21 days	10.49	[1.68–65.5; 95% CI]	N/A	N/A	1. MACE
									2. All-cause Mortality
Fan et al. (22)	107	cTnI:= 14 (5–97)	N/A	32 days	N/A	N/A	2.66	[1.44–4.92; 95% CI]	1. All-cause Mortality
		Non-severe group: 7 (2–16) folds							
		Severe group: 48 (10,191) folds							
Dubey et al. (29)	35	Short-term survival group:	N/A	42 days	N/A	N/A	N/A	N/A	N/A
		1,316 ng/L (858–2,490)							
		Intermediate-term survival group 180 ng/L							
		(32–37)							
		Long-term survival group:							
		233 ng/L (36–684)							

MACE, major adverse cardiovascular events; HR, hazard ratio; CI, confidence interval; CV, cardiovascular; cTnI, cardiac troponin I; cTnT, cardiac troponin T; OS, overall survival; N/A, not applicable/not available; ESC, european society of cardiology; ng/L, nanograms per liter; ULN, upper limit of normal.

### Risk of bias and certainty of evidence

**Figure 7:**
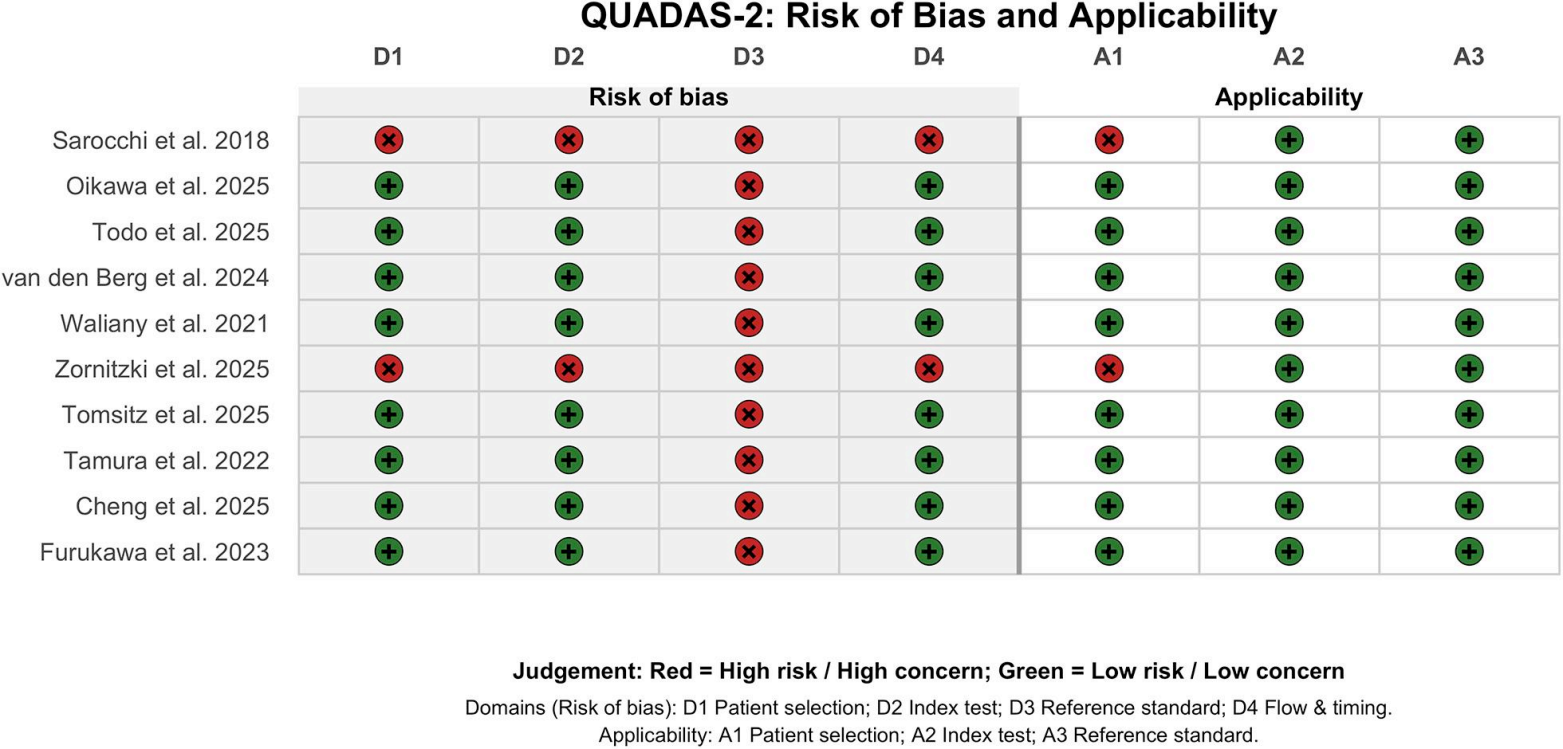
QUADAS-2 risk-of-bias summary for diagnostic/screening studies. Traffic-light plot summarizing risk of bias and applicability concerns for included diagnostic and screening studies assessed with QUADAS-2. Domains include patient selection, index test, reference standard, and flow/timing, rated as low, unclear, or high risk. The reference standard domain was frequently rated high risk where myocarditis definitions incorporated troponin, introducing incorporation bias. QUADAS-2, Quality Assessment of Diagnostic Accuracy Studies-2.

**Figure 8:**
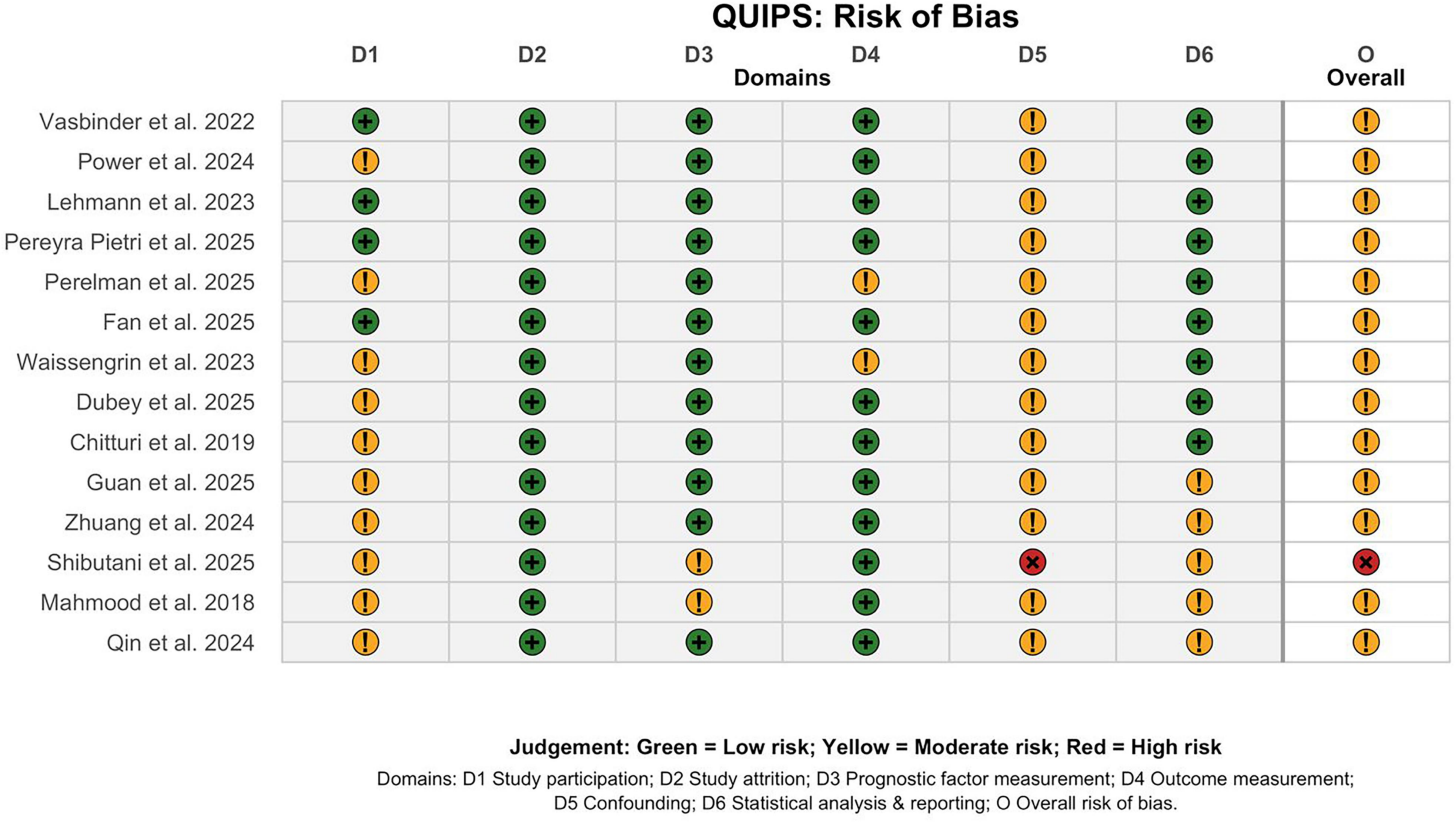
QUIPS risk-of-bias summary for prognostic studies. Traffic-light plot summarizing risk of bias across included prognostic studies using the QUIPS tool. Domains include study participation, study attrition, prognostic factor measurement, outcome measurement, study confounding, and statistical analysis/reporting, rated as low, moderate, or high risk. Confounding was the most common concern due to limited adjustment for baseline cardiovascular and cancer-related factors in several cohorts. QUIPS, Quality In Prognostic Studies.

Risk of bias and applicability for diagnostic studies were assessed using the QUADAS-2 tool (Figure 7), while prognostic studies were evaluated using the QUIPS instrument (Figure 8).

Certainty of evidence for each outcome was assessed using the GRADE framework (Table 4). Diagnostic studies were predominantly judged to be at high overall risk of bias, driven mainly by the reference standard domain. In most studies, myocarditis diagnosis relied on guideline- or consensus-based criteria that incorporated troponin elevation, resulting in incorporation bias and limiting independent evaluation of troponin as an index test. Patient selection and flow and timing domains were generally at low risk of bias, particularly in prospective surveillance cohorts with predefined inclusion criteria and serial biomarker assessment. Applicability concerns were low overall, reflecting contemporary cardio-oncology practice. Consequently, the certainty of evidence for diagnostic performance was rated as very low.

**Table 4:** GRADE certainty of evidence assessment.

**Outcome**	**Initial Certainty**	**Risk of Bias**	**Indirectness**	**Inconsistency**	**Imprecision**	**Publication Bias**	**Other Factors**	**Final Quality**
**Diagnostic Performance**	Low	Serious	Not serious	Serious	Serious	Suspected	Large effect (high negative predictive value)	Very low
**Major adverse cardiovascular events (MACE)**	Low	Serious	Not serious	Serious	Not serious	Suspected	Large effect	Low
**Mortality**	Low	Serious	Not serious	Not serious	Not serious	Suspected	Dose–response gradient; plausible residual confounding	Moderate

Prognostic studies demonstrated low to moderate risk of bias overall. Most studies showed low risk in domains related to study participation, attrition, prognostic factor measurement, and outcome assessment. Confounding was the most frequent source of bias, with several studies lacking comprehensive multivariable adjustment. Nevertheless, effect estimates were consistent and statistical analyses were generally appropriate.

Accordingly, the certainty of evidence for associations between troponin elevation and major adverse cardiovascular events was rated as low, while certainty for mortality outcomes was rated as moderate, supported by consistency of findings, absence of serious imprecision, and evidence of a dose–response relationship.

## Discussion

### Principal findings

This systematic review and meta-analysis provides the most comprehensive evaluation to date of the diagnostic and prognostic utility of troponin in ICI-associated myocarditis. By synthesizing quantitative evidence from 4 diagnostic studies and 11 prognostic studies, this review represents the largest pooled assessment of troponin in signaling ICI-myocarditis and stratifying cardiovascular and mortality risks in ICI-treated patients.

Across diagnostic studies, elevated troponin levels were associated with confirmed ICI-related myocarditis, reflected in a pooled log odds ratio of 4.14, highlighting its importance as an early diagnostic biomarker of ICI-associated myocarditis in adult cancer patients undergoing ICI therapy. Clinically, this association suggests that troponin elevation in ICI-treated patients should prompt urgent evaluation and management for potential myocarditis, particularly when accompanied by symptoms, electrocardiographic abnormalities, imaging findings, or rising biomarker levels.

In prognostic analyses, elevated troponin levels were associated with a markedly increased risk of major adverse cardiovascular events (MACE) (pooled HR 6.14, 95% CI 3.61–10.45) and with higher mortality (pooled HR 1.95, 95% CI 1.36–2.81), underscoring troponin's role not only in diagnosis but also in risk stratification and outcome prediction following ICI therapy.

### Diagnostic signal and interpretation

Cardiac troponin elevation is a cornerstone of the diagnostic evaluation of ICI–associated myocarditis and plays an increasingly important role in risk stratification during ICI therapy (5). However, troponin elevation is common in this population and reflects a spectrum of myocardial and systemic injury, underscoring the need for careful contextual interpretation. In this systematic synthesis, we demonstrate that cardiac troponin elevation is a sensitive marker of ICI-associated myocardial injury but lacks specificity as a standalone diagnostic test for myocarditis. Across diagnostic, prognostic, and surveillance studies, troponin elevation consistently identified patients at increased cardiovascular risk, including those without adjudicated myocarditis, supporting its role as a risk-stratification rather than purely diagnostic biomarker (6–10). Importantly, the prognostic value of troponin was strongly influenced by magnitude, trajectory, and subtype, with persistent elevation, particularly of cardiac troponin T, reflecting ongoing immune-mediated cardiomuscular injury and worse outcomes (5, 13). Together, these findings support an integrated, longitudinal approach to troponin interpretation that emphasizes clinical context and dynamic change over isolated thresholds in patients receiving ICI therapy.

Important differences between cardiac troponin I (cTnI) and cardiac troponin T (cTnT) have direct implications for diagnostic sensitivity and interpretation. Most reported cases of ICI myocarditis have been identified using cTnI, reflecting its widespread availability and incorporation into contemporary diagnostic frameworks. cTnI has traditionally been regarded as more cardiac-specific than cTnT and has therefore been favored as the primary biomarker in this setting (5). Emerging evidence, however, challenges the adequacy of cTnI as a standalone exclusionary marker. In reported cases of ICI myocarditis diagnosed despite negative troponin testing, the assay used was consistently cTnI (5). Observational cohorts further suggest that a clinically meaningful subset of patients with adjudicated ICI myocarditis demonstrate isolated cTnT elevation in the absence of cTnI elevation at presentation (5, 13). This discordance highlights potential limitations of relying on a single troponin subtype when evaluating suspected immune-mediated myocardial injury.

Beyond troponin, creatine kinase–MB (CK-MB) has been employed historically as a marker of myocardial injury and retains a complementary role in suspected ICI-associated myocarditis, although it is less cardiospecific than troponin and can be elevated by concomitant skeletal-muscle injury (37, 38). Given that ICI-associated myocarditis frequently overlaps with myositis particularly in the setting of combination immunotherapy concurrent measurement of total creatine kinase (CK) and CK-MB alongside troponin enhances diagnostic discrimination of the overlap phenotype and identifies patients at increased risk of fulminant disease (20, 39). In the Lehmann et al. cohort, simultaneous elevation of CK, CK-MB, and troponin defined a cardiomuscular phenotype with worse outcomes than isolated troponin elevation, and disproportionate increases in CK-MB relative to total CK have been described as supportive though non-specific features of myocardial involvement (20). We therefore consider troponin as the primary biomarker for early detection of ICI-associated myocardial injury, with CK and CK-MB serving as adjunctive markers that help characterize disease phenotype, identify myositis–myocarditis overlap, and inform risk stratification rather than functioning as standalone diagnostic tools.

### Prognostic value for Major adverse cardiovascular events and mortality

Major adverse cardiovascular events (MACE) have emerged as an important and clinically consequential complication in patients undergoing ICI therapy. Although ICIs have transformed oncologic outcomes, cardiovascular immune-related adverse events contribute disproportionately to early morbidity and mortality, with a substantial proportion occurring within the first weeks to months after treatment initiation. Observational cohorts and pharmacovigilance analyses consistently demonstrate that MACE—including arrhythmias, cardiomyopathy, pericardial disease, acute coronary syndromes, and cardiovascular death—cluster early during ICI exposure, underscoring the need for reliable strategies for early risk stratification and detection (30, 31, 34).

Among the spectrum of ICI-related cardiovascular toxicities, myocarditis represents the most severe and prognostically significant entity. Despite its relatively low incidence, ICI-associated myocarditis carries a markedly higher risk of MACE and early mortality compared with other cardiovascular immune-related adverse events and with myocarditis of non-ICI etiologies (17, 30, 31). Reported rates of MACE and cardiovascular death are several-fold higher and occur over a compressed time course, reflecting an aggressive clinical phenotype rather than progressive structural heart disease. Consequently, myocarditis has become a central focus of cardio-oncology surveillance strategies and a critical determinant of cardiovascular outcomes in ICI-treated patients (17, 31).

Importantly, the risk and severity of ICI-associated myocarditis are not uniform across regimens. Combination immune checkpoint blockade particularly anti–CTLA-4 plus anti–PD-1/PD-L1 confers a disproportionate cardiovascular risk compared with monotherapy (17, 31, 34). In the seminal multicenter registry by Mahmood and colleagues, the prevalence of myocarditis was approximately 2.4% with combination anti–PD-1/anti–CTLA-4 therapy, compared with 0.5% with anti–PD-1 monotherapy, with combination regimens also associated with earlier onset, greater troponin elevation, more frequent overlap with myositis and myasthenia gravis, and a higher incidence of fulminant courses and MACE (17). Pharmacovigilance analyses corroborate these findings, with a reporting odds ratio of approximately 4 for myocarditis with dual vs. single ICI exposure, and the early-onset, concurrent myocarditis–myositis phenotype consistently associated with the worst outcomes (39, 40). Mechanistically, simultaneous blockade of CTLA-4 and PD-1 amplifies T-cell activation and antigen-driven cardiomuscular inflammation, consistent with preclinical models in which combined Ctla4/Pdcd1 haploinsufficiency reproduces the clinical and pathological features of ICI myocarditis (32, 33). Patients receiving combination ICI therefore represent a high-risk subgroup in whom troponin elevation should be interpreted with a particularly low threshold for comprehensive cardiovascular evaluation and consideration of early immunosuppression.

Beyond its diagnostic utility, troponin elevation in ICI-associated myocarditis carries substantial prognostic significance, particularly for MACE and early mortality. In this context, troponin elevation functions not merely as a marker of myocardial injury, but as an integrated indicator of disease severity and overall cardiovascular risk. Across observational and registry-based studies, troponin elevation has been consistently associated with adverse outcomes, supporting its role as a prognostic rather than purely diagnostic biomarker in this setting (17, 26, 28, 29).

Clinically, troponin elevation has been consistently associated with higher rates of MACE, including malignant arrhythmias, conduction disturbances, hemodynamic instability, and cardiovascular death. Importantly, these events frequently occur despite preserved left ventricular systolic function, underscoring that adverse outcomes are not reliably captured by conventional functional assessments alone. In contrast, electrocardiography and clinical symptoms are nonspecific, echocardiography may remain normal early in the disease course, and cardiac magnetic resonance imaging particularly late gadolinium enhancement may have limited early prognostic discrimination in ICI-associated myocarditis, especially when imaging is performed early or follow-up duration is short (17, 31, 35). Against this backdrop, troponin provides a quantifiable and dynamic biomarker that offers superior early prognostic insight when structural or functional assessments are equivocal.

Troponin elevation also stratifies mortality risk beyond structural myocardial injury. Persistent or marked elevations identify patients at particularly high risk for early death, even in the absence of overt systolic dysfunction (17, 26, 32, 33). Accordingly, troponin elevation in ICI-associated myocarditis should be interpreted as a high-risk prognostic marker rather than a binary diagnostic threshold, reinforcing its central role in identifying patients at greatest risk for MACE and early mortality.

Although troponin elevation is a strong predictor of MACE and mortality, baseline patient characteristics may further modulate overall risk. Advanced age and adverse cardiometabolic profiles, particularly obesity and pre-existing cardiovascular disease, are frequently observed in affected patients and may amplify the prognostic significance of troponin elevation by reflecting reduced physiologic reserve (25, 34). In this setting, troponin elevation likely captures not only myocardial injury but also vulnerability to rapid clinical deterioration and early death.

### Clinical implications

Collectively, these findings support a contextualized approach to troponin interpretation during ICI therapy. Given its high sensitivity but limited specificity, troponin is best positioned as a surveillance biomarker and triage tool within an integrated cardio-oncology framework that incorporates clinical assessment, electrocardiography, cardiac imaging, and longitudinal monitoring (5, 6, 36).

Routine cardiac troponin surveillance in patients receiving ICI is clinically important despite the low incidence of ICI-associated myocarditis, as early myocardial injury is often clinically silent and delayed recognition is associated with high morbidity and mortality; moreover, troponin elevation after ICI initiation identifies patients at increased risk of major adverse cardiovascular events and death, even in the absence of confirmed myocarditis. Current cardio-oncology guidance most notably the 2022 ESC Cardio-Oncology Guidelines recommends baseline cardiac troponin measurement in all patients initiating ICI therapy (class I), with serial measurement before doses 2, 3, and 4 and every three doses thereafter (class IIa), with intensified monitoring in higher-risk patients such as those receiving dual ICI therapy, those with prior cardiotoxic exposure, pre-existing cardiovascular disease, or concomitant non-cardiac irAEs (27, 38, 41). Within this surveillance context, isolated low-level troponin elevations should prompt clinical vigilance rather than reflexive interruption of cancer therapy, particularly in the absence of corroborating clinical, electrocardiographic, or imaging findings (24). Conversely, persistent elevations, rising trajectories, or the development of cardiac symptoms should lower the threshold for comprehensive cardiovascular evaluation (35). In patients with suspected or confirmed ICI-associated myocarditis, sustained troponin elevation identifies those at highest risk of early adverse events and mortality and may justify intensified monitoring and early escalation of immunosuppressive strategies (36).

In practical terms, no single universally validated absolute troponin cutoff for ICI-induced myocarditis exists, reflecting heterogeneity of assays, populations, and pre-test probabilities (5, 37, 38). We therefore suggest operationalizing troponin interpretation around four practical scenarios: (i) baseline troponin elevation should prompt evaluation for pre-existing cardiac disease and informed shared decision-making before initiating ICI; (ii) isolated low-level elevation during therapy (e.g., values just above the assay-specific 99th-percentile upper reference limit, without clinical, ECG, or imaging abnormalities) should trigger repeat measurement within 24–72 h, 12-lead ECG, transthoracic echocardiography, and consideration of natriuretic peptides, rather than reflexive ICI interruption; (iii) rising trajectory or relative change (≥20%–50%) on serial measurement should be regarded as a high-risk pattern that prompts urgent multidisciplinary cardio-oncology evaluation, ICI interruption, and a low threshold for cardiac MRI and/or initiation of corticosteroids; and (iv) overt elevation with supportive clinical, ECG, or imaging findings should be treated as presumed ICI-associated myocarditis pending adjudication, with prompt ICI cessation and initiation of high-dose corticosteroids while alternative etiologies (acute coronary syndrome, viral myocarditis) are excluded (37, 38, 41). Across all scenarios, integrated assessment combining troponin trajectory, ECG, imaging, and concurrent CK/CK-MB measurement provides more reliable risk stratification than any single cutoff.

### Recommendations

Current diagnostic frameworks incorporate troponin as a core criterion for ICI-associated myocarditis. However, they do not fully address its prognostic significance across the broader ICI-treated population or account for dynamic changes over time. Our synthesis extends existing guidance by emphasizing that serial troponin assessment provides clinically meaningful information beyond diagnosis, particularly for risk stratification and treatment monitoring.

Pending prospective validation, we suggest that future iterations of cardio-oncology guidelines should: (i) endorse risk-stratified troponin surveillance with intensified monitoring in patients receiving combination ICI or with established cardiovascular risk factors; (ii) standardize the operational definition of a clinically meaningful troponin change (absolute and relative) during ICI surveillance; (iii) explicitly differentiate the diagnostic interpretation of cTnI vs. cTnT, and integrate CK and CK-MB to identify the myocarditis–myositis overlap phenotype; and (iv) align thresholds for ICI interruption and corticosteroid initiation with the integrated clinical-biomarker phenotype rather than with isolated troponin values (37–41).

## Limitations

Our findings should be interpreted considering several limitations. The available evidence is largely retrospective and observational, with relatively small sample sizes in many cohorts, introducing potential selection bias and limiting generalizability. Diagnostic studies were frequently conducted in referral settings with a high pretest probability of myocarditis, which may inflate diagnostic associations compared with unselected ICI–treated populations. In addition, only English-language studies were included, which may have introduced language bias and limited the comprehensiveness of the evidence base. Substantial heterogeneity existed in troponin assays and thresholds, including use of cardiac troponin I vs. troponin T, conventional vs. high-sensitivity platforms, institution-specific cutoffs, and variable timing of sampling ranging from routine surveillance to symptom-driven testing. This heterogeneity precluded identification of standardized diagnostic thresholds and may influence both diagnostic and prognostic effect estimates. Diagnostic pooling relied primarily on odds ratios due to inconsistent reporting of sensitivity, specificity, and uniform adjudication criteria, limiting inference regarding test performance. Prognostic associations between troponin elevation and adverse outcomes may be confounded by baseline cardiovascular risk and illness severity, and outcome definitions varied across studies. Finally, long-term cardiovascular outcomes remain insufficiently characterized, and variability across clinical settings, reflected by wide prediction intervals in some analyses, underscores the need for prospective studies using standardized surveillance protocols, predefined diagnostic pathways, and extended follow-up to refine escalation thresholds and optimize troponin-guided management in patients receiving ICIs.

## Conclusion

In patients treated with ICIs, troponin elevation is associated with ICI-related myocarditis and identifies individuals at increased risk of adverse cardiovascular outcomes and mortality, but false-positive elevations are common and limit its specificity as a standalone diagnostic test. Across heterogeneous study designs and assays, these findings support the role of troponin as a readily available biomarker for early clinical evaluation and risk stratification in this high-risk population.

However, variability in assay type, diagnostic thresholds, and timing of measurement currently limits the ability to define standardized clinical cutoffs or uniform diagnostic pathways. Troponin elevation should therefore be interpreted within the broader clinical context and integrated with electrocardiography, cardiac imaging, and clinical judgment rather than used in isolation.

Future prospective studies with standardized troponin measurement, clearly defined diagnostic criteria, and prespecified outcome assessment are needed to refine its diagnostic utility and to determine how troponin-guided strategies can be optimally implemented within cardio-oncology surveillance and risk-stratification pathways.

## Perspectives

### Competency in medical knowledge and patient care

In patients treated with immune checkpoint inhibitors, elevation of cardiac troponin is associated with immune checkpoint inhibitor–associated myocarditis and identifies individuals at substantially increased risk of major adverse cardiovascular events and early mortality. Troponin should be interpreted as a sensitive marker of myocardial injury and a powerful prognostic biomarker rather than as a standalone diagnostic test. Serial troponin assessment during immune checkpoint inhibitor therapy provides clinically meaningful information for early detection, risk stratification, and treatment monitoring, particularly when interpreted in conjunction with clinical assessment, electrocardiography, and cardiac imaging. Persistent or rising troponin levels identify a high-risk subgroup that may benefit from intensified cardiovascular surveillance and early escalation of immunosuppressive therapy.

### Translational outlook

Future prospective studies are needed to define standardized troponin surveillance strategies in patients receiving immune checkpoint inhibitors, including optimal timing, frequency, and assay selection. Research should focus on establishing validated diagnostic thresholds, integrating troponin trajectories into risk prediction models, and determining whether troponin-guided management algorithms improve cardiovascular outcomes without compromising oncologic efficacy. Multidisciplinary cardio-oncology collaboration will be essential to translate biomarker-driven strategies into routine clinical practice and to refine personalized approaches for prevention, early detection, and treatment of immune checkpoint inhibitor–related cardiotoxicity.

## Data Availability

The original contributions presented in the study are included in the article/Supplementary Material, further inquiries can be directed to the corresponding author.

## Appendix 1

Detailed Search Strategy

Search Structure

**Table:**

BLOCK A	AND	BLOCK B
Immune Checkpoint Inhibitors		Cardiac Biomarkers/Troponin

1. Block A — Immune Checkpoint Inhibitors

This block captures the exposure of interest: immunotherapy agents that modulate immune checkpoints. Terms within Block A are combined using OR to maximise sensitivity. The block is organised into four sub-categories:

1.1 Controlled vocabulary (MeSH)

MeSH headings provide standardised indexing and retrieve indexed articles regardless of how the author described the intervention:

**Table:**

MeSH Term	Scope/Rationale
Immune Checkpoint Inhibitors	Broad class-level MeSH for all ICI agents
Programmed Cell Death 1 Receptor	PD-1 receptor on T-cells; targeted by anti-PD-1 antibodies
Programmed Cell Death 1 Ligand 1 Protein	PD-L1 protein on tumour/immune cells; targeted by anti-PD-L1 antibodies

1.2 Class/Pathway Terms (free text)

Broader conceptual terms used by authors to describe this class of therapy:

checkpoint inhibitor* — wildcard captures “checkpoint inhibitor”, “checkpoint inhibitors”, “checkpoint inhibition”

immune checkpoint* — captures “immune checkpoint therapy”, “immune checkpoint blockade”, etc.

ICI — common abbreviation used in recent clinical and research literature

1.3 Target-Specific Terms (free text)

Terms describing the specific molecular targets of ICI therapy:

PD-1/PD1 — Programmed Death-1 receptor

PD-L1/PDL1 — Programmed Death Ligand-1

CTLA-4 — Cytotoxic T-Lymphocyte-Associated protein 4

1.4 Individual Drug Names (free text)

2. Block B — Cardiac Biomarkers/Troponin

This block captures the outcome of interest: cardiac troponin and related biomarker measurements used to detect myocardial injury. Terms are combined with OR.

2.1 Controlled Vocabulary (MeSH)

**Table:**

MeSH Term	Scope
Troponin	Broad MeSH capturing all troponin subtypes
Troponin I	Cardiac isoform I; preferred in clinical practice
Troponin T	Cardiac isoform T; widely used in hs-cTn assays

2.2 Free-Text Biomarker Terms

Free-text terms supplement MeSH to capture articles that may not be fully indexed or use terminology not reflected in MeSH:

troponin — generic term; captures all mentions of troponin in titles/abstracts

cardiac troponin — specifies cardiac origin, distinguishing from skeletal muscle troponin

troponin I — isoform-specific term

troponin T — isoform-specific term for the T subunit

hs-cTn* — wildcard abbreviation covering hs-cTnI, hs-cTnT, hs-cTn; refers to high-sensitivity cardiac troponin assays

high-sensitivity troponin — full phrase for hs assay; hyphenated form

high sensitivity troponin — same concept, unhyphenated variant; both captured for completeness

cardiac enzym* — broader term capturing cardiac enzyme elevation in general; wildcard covers “enzyme”, “enzymes”, “enzymatic”
